# Topographical and histological analysis of keratinized mucosal grafts removal techniques an ex-vivo study in porcine mandibles

**DOI:** 10.1038/s41598-024-58559-w

**Published:** 2024-06-06

**Authors:** Kleber Vinícius Rodrigues dos Santos, José Luiz Rodrigues Leles, Virgílio Moreira Roriz, Cláudio Rodrigues Leles

**Affiliations:** 1 School of Dentistry, Sao Paulo University, Av. Do Café-Subsetor Oeste-11, Ribeirão Preto, SP 14040-904 Brazil; 2 School of Dentistry, Paulista University, Rodovia BR 153, Km 503, s/n Fazenda Botafogo, Goiânia, GO 74215060 Brazil; 3 School of Dentistry, Federal University of Goiás, Av. Universitária, s/n-Setor Leste Universitário, Goiânia, GO 74605-020 Brazil

**Keywords:** Keratinized mucosa grafts, Mucotome, Scanning electron microscope, Swine jaw, Biological techniques, Medical research

## Abstract

The aim of this study was to assess the surface and tissue quality of keratinized mucosa grafts (KMG) obtained using the conventional scalpel and mucotome techniques. This was an experimental in vitro/ex vivo study involving six porcine hemi-mandibles. Specimens were harvested using both the mucotome and conventional scalpel techniques, with randomization determining the choice of technique for tissue removal. The specimens were prepared following predefined laboratory protocols and subsequently subjected to optical microscopy for evaluating epithelial and connective tissue and scanning electron microscopy for topographical and 3D profilometry analysis. Tissues harvested using the mucotome exhibited a linear base and uniform thickness, along with the presence of submucosa and fibrous connective tissue, all of which are ideal for graft success. Differences in the surface characteristics of specimens obtained through the two techniques were observed during a comparative analysis of images obtained through both microscopy types. KMG obtained using the mucotome technique displayed greater uniformity and reduced undesirable cell presence compared to the scalpel technique, thereby enhancing the likelihood of success in soft tissue graft surgical procedures. This study provides valuable insights to oral healthcare professionals and may contribute to future research aimed at achieving more successful surgeries, shorter postoperative recovery times, reduced discomfort, and an overall more positive patient experience.

## Introduction

A graft is defined as a piece of tissue with viable cells, taken from a donor area and placed in a recipient area, obtained and used in the same surgical procedure, with the intention of reconstructing the recipient area. Grafts can be autogenous (originating from the individual themselves), allogeneic (originating from individuals of the same species), xenogeneic (originating from individuals of different species), or can also be composed of biomaterials based on collagen matrix^1^. According to Misch^2^, autogenous grafts are considered the gold standard compared to others. The types of gingival grafts that have been more widely used and well-documented in the literature for gaining keratinized tissue are free gingival graft (FGG) and connective tissue graft (CTG). In addition, acellular dermal matrix, which is a biomaterial, can also be used^3^. These have demonstrated a statistically significant increase in the amount of keratinized tissue obtained^4–7^.

Among the types of grafts used to obtain a satisfactory width of keratinized tissue, free gingival graft (FGG) is frequently employed. However, this technique, initially described by Sullivan and Atkins^6^, presents some limitations, including graft shrinkage between 12 and 58% during the healing period^8–14^, esthetics limitations^14,15^ and postoperative pain at both donor and recipient sites^16–18^. The FGG technique is still considered the approach of choice for soft tissue and keratinized mucosa augmentation around teeth and peri-implant regions. In contrast, the CTG provides greater predictability in cases of complete root coverage and dehiscence, yielding favorable esthetics outcomes^19^. Subsequently, Duarte and Castro^20^ proposed a modification of the technique where the graft is first secured and then covered by a partial-thickness flap. The advantages of this approach include reduced graft shrinkage, improved coloration, and reduced postoperative pain and discomfort reported by patients.

Autogenous connective tissue grafts have been used in numerous clinical procedures, such as root coverage in cases of gingival recession^20^, ridge preservation or augmentation, papillae reconstruction, and implant recipient area preparation^21^. This procedure promotes increased volume and keratinized gingiva, and is associated with significantly less bone loss in peri-implant regions^22–24^. Furthermore, keratinized tissue facilitates restorative procedures, enhances esthetics, and enables better oral hygiene maintenance with less discomfort and irritation for patients^25–27^.

For the removal of soft tissue grafts, a scalpel blade is commonly used^4,7,28–32^. Another alternative for graft harvesting is using a mucotome—a contra-angle handpiece with a cutting blade driven by an electric motor. It offers high precision with an oscillating blade and is used as a handpiece, capable of producing smooth grafts of uniform thickness^33^. According to the manufacturer’s manual, Nouvag^34^, the mucotome’s cutting blade has a thickness of 0.5 mm and a width of 6 mm. Furthermore, the graft removal technique does not predominantly depend on the operator’s experience and skill, as in the conventional technique, due to the standardization of the thickness and width of the cuts made by the device.

This is a primary study, and there is no scientific literature that compares, at the microscopic level, the surface of keratinized mucosa grafts (KMG) in terms of topography, roughness, uniformity, and the types of cells present, when removed using the conventional technique and the mucotome technique. The aim was to assess whether KMGs removed using the conventional scalpel, compared to the mucotome technique, exhibit variations in surface roughness and uniformity. The null hypothesis is that grafts removed using the mucotome exhibit greater surface uniformity and better tissue quality of the specimen compared to the conventional technique.

## Objective

This study aims to evaluate the surface and tissue quality of keratinized mucosal grafts (KMGs) obtained using the mucotome and conventional scalpel techniques. It involves the assessment of KMGs obtained from pig mandibles (*Sus scrofa domesticus*) using both techniques and employing optical microscopy and scanning electron microscopy (SEM). Additionally, the study seeks to compare the topographical features and 3D profilometry of KMGs acquired via these techniques through SEM analysis.

## Material and methods

**Figure 1:**
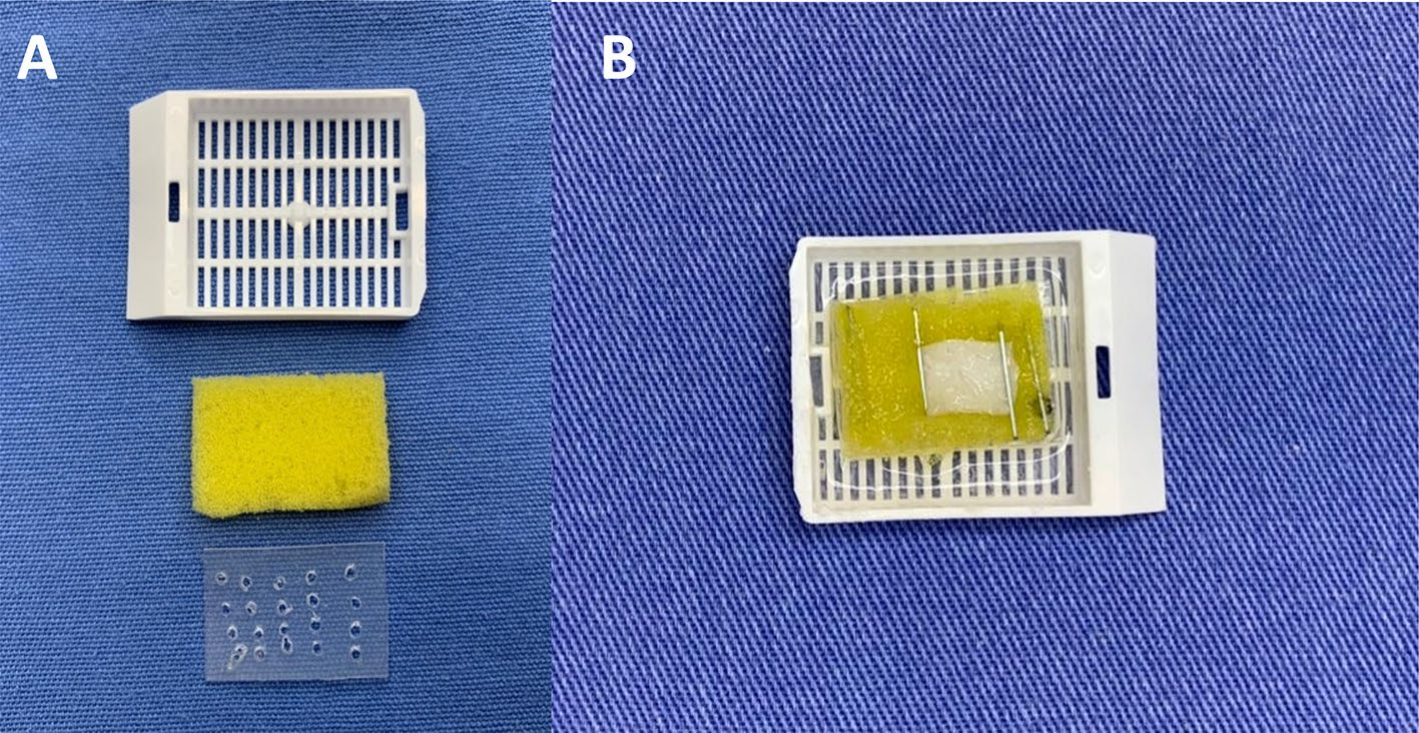
Bracket for supporting specimens during SEM analysis.

A pilot project aimed to test the proposed methodology and reduce bias. Fresh specimens were removed, fixed in a storage and reading device, and placed in 10% formaldehyde for preservation. The foam and plastic device was designed to maintain specimen moisture and adequate surface quality during SEM analysis (Fig. 1). The pilot project allowed the development of a methodology suitable for the research context and determined the sample size. These samples were then analyzed using SEM, resulting in improved image quality for comparing the graft removal techniques.

This research is an experimental in-vitro/ex-vivo study. It commenced after approval by the Ethics Committee for Animal Use in Research (CEUA UFG) under the number MB 029/21. The datasets used and/or analysed during the current study available from the corresponding author on reasonable request.

Six hemi-mandibles from the Suidae family, *Sus* genus, *S. domesticus* species, Landrace breed, and scientific name *Sus scrofa domesticus* were utilized. The specimens presented a favorable condition of keratinized tissue for removal on the inner face in the anterior region of the mandible. This area was chosen for specimen removal due to its higher quantity and superior tissue quality compared to these pieces’ middle or posterior regions. The swine mandibles were obtained from a butcher with updated registration and licensed from the health surveillance agency in Goiânia, Goiás, Brazil. They were stored in a thermal box with ice to preserve the specimens and facilitate transportation to the location for tissue removal procedures. The time between obtaining the mandibles and the commencement of the procedure was 1 h. To ensure greater precision, a specialized and previously trained dentist carried out the removal of specimens from the mandibles, providing the same conditions for graft removal using both the mucotome and conventional techniques. The randomization process was conducted through a random drawing to determine which technique would be used for KMG removal (conventional technique or mucotome technique) and to specify the region of graft removal from the inner anterior face of the mandible for analysis via optical microscopy or SEM (anterior or posterior region of the graft).

### Specimen acquisition and preparation

A template made of cardboard from a 4-0 nylon suture packaging, measuring 6 × 16 mm, was used to standardize sample dimensions. This template simulated the typical guides for graft surgery in the dental clinic. The template was applied to the entire sample to outline the size of the specimens removed using different techniques. The template’s edges were delineated using a #15 blade to serve as a guide for specimen removal from the donor area. After delineation, specimen removal began according to the pre-established randomization.

### Mucotome technique

**Figure 2:**
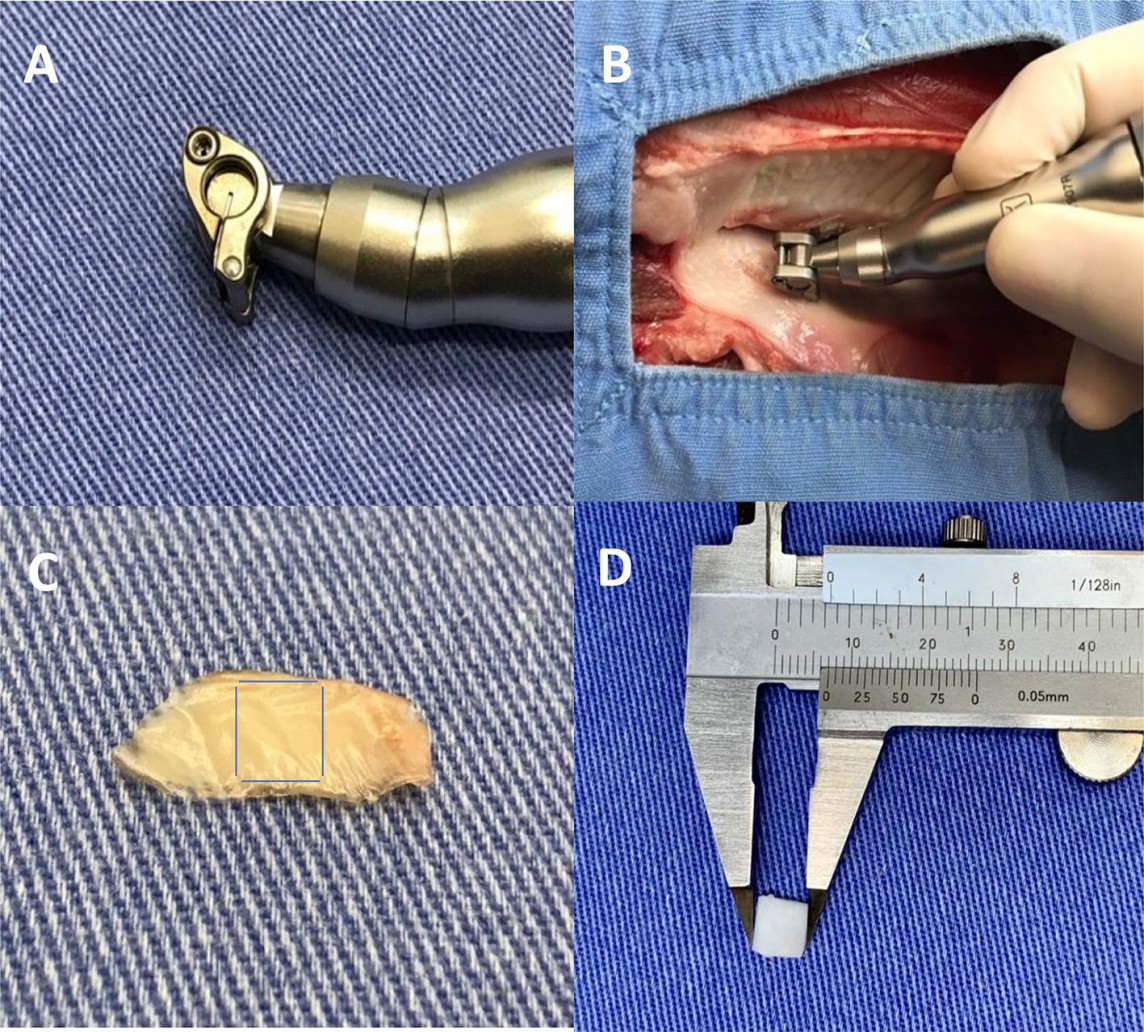
(**A**) Mucotome, handpiece; (**B**) graft removal using the mucotome; (**C**) Removed specimen with delimitations of the sectioned area in the middle third of sample with 0.5 mm of thickness; (**D**) measurement of the size of the removed and already sectioned specimen.

**Figure 3:**
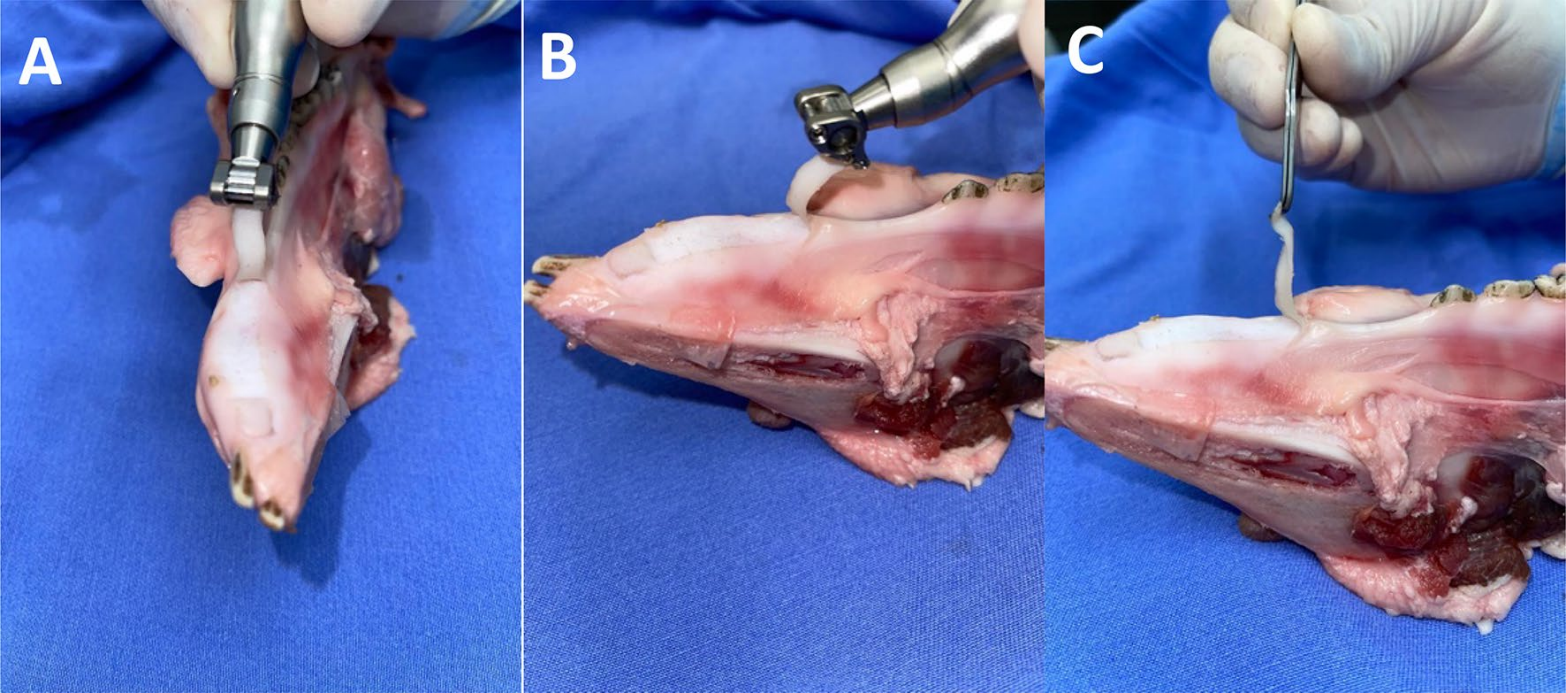
Removal of KMC using the mucotome.

The procedure employed a mucotome (Mucotome Ref. 1970, Nouvag, Goldach, Switzerland) (Fig. 2) with a blade width of 6 mm and a cutting depth of 0.5 mm. The mucotome was attached to an electric implant motor (NeoSurg Pro, Curitiba, Paraná, Brazil) by Neodent, operating at a speed of 1000 rotations per minute (rpm) and a torque of 50 N/cm^2^. According to the manufacturer’s Nouvag manual, the mucotome can operate at up to 8000 rpm. However, during the pilot project, standardization of 1000 rpm and 50 N/cm^2^ was chosen for specimen removal to ensure that tissues would be removed with integrity. The removal of the three specimens was carried out with a mild pressure of approximately 20–25 N/cm^2^ on the tissue, using a constant anteroposterior motion and a #15C blade was employed to completely separate the specimen from the porcine mandible. There was no macroscopically excessive tissue in any of the three specimens, and no treatment of the inner surface of the specimen was performed. Subsequently, the template made of cardboard with dimensions of 6 × 16 mm was used to outline the sample’s size. This sample was then divided into two parts (anterior and posterior regions), resulting in six parts measuring 6 × 8 mm, as depicted in Figs. 2 and 3.

### Conventional scalpel technique

**Figure 4:**
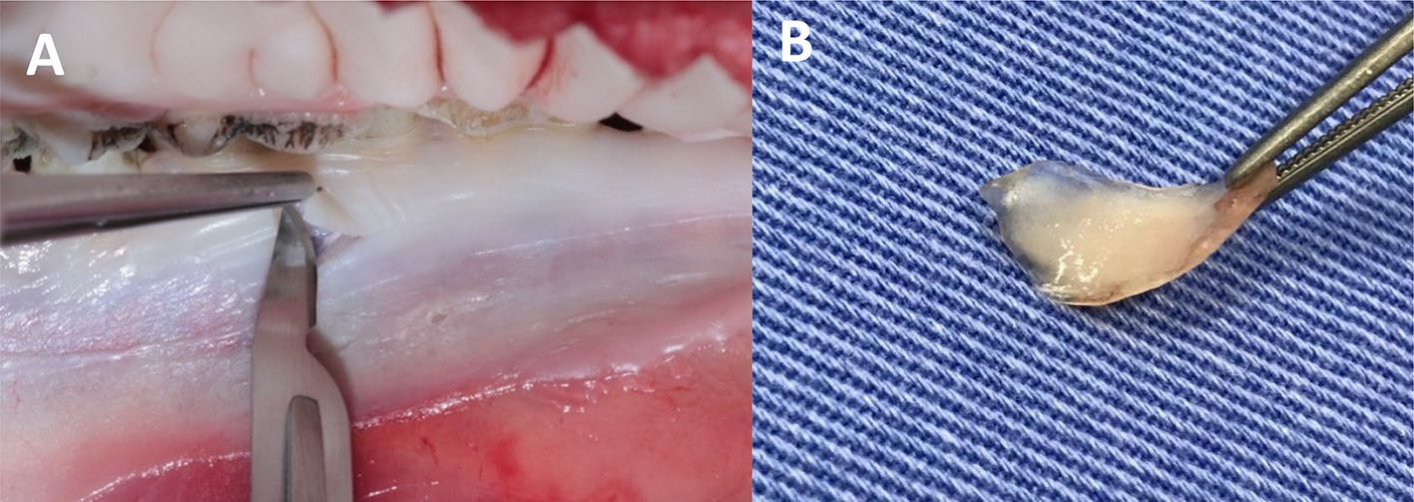
Graft removal by conventional technique. (**A**) Scalpel blade making the tissue incision. (**B**) Tissue removed by the scalpel blade.

**Figure 5:**
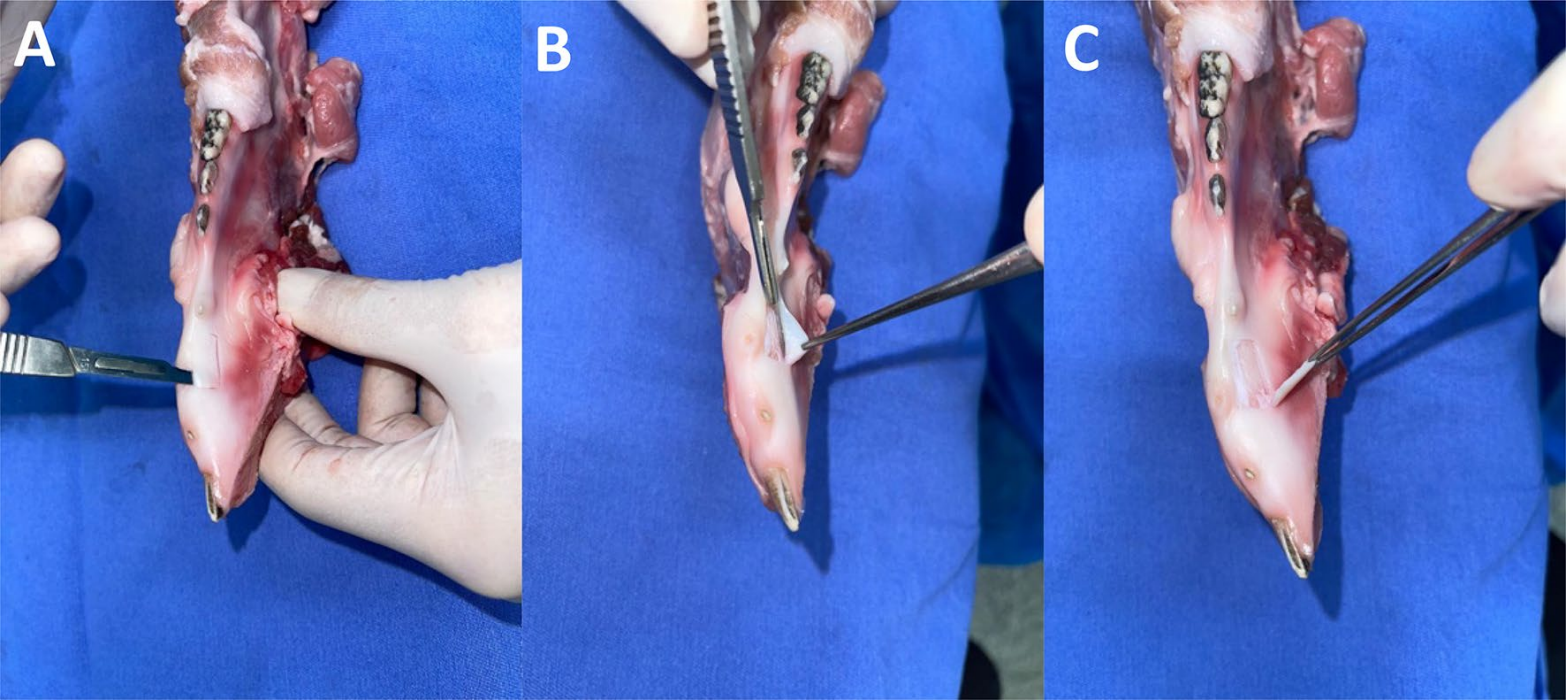
Graft removal using a scalpel.

For the conventional technique, a new No. 15C scalpel blade (Solidor, Anhui Easyway Medical, China) was used with an initial incision perpendicular to the specimen, followed by an incision at an angle between 30º and 45º. After this initial incision, the scalpel blade was directed parallel to the epithelial surface, separating the underlying connective tissue and obtaining a laminar rectangular piece of epithelialized tissue with dimensions of 16 mm (length), 6 mm (width) and 2 mm (thickness) (Figs. 4 and 5).

**Figure 6:**
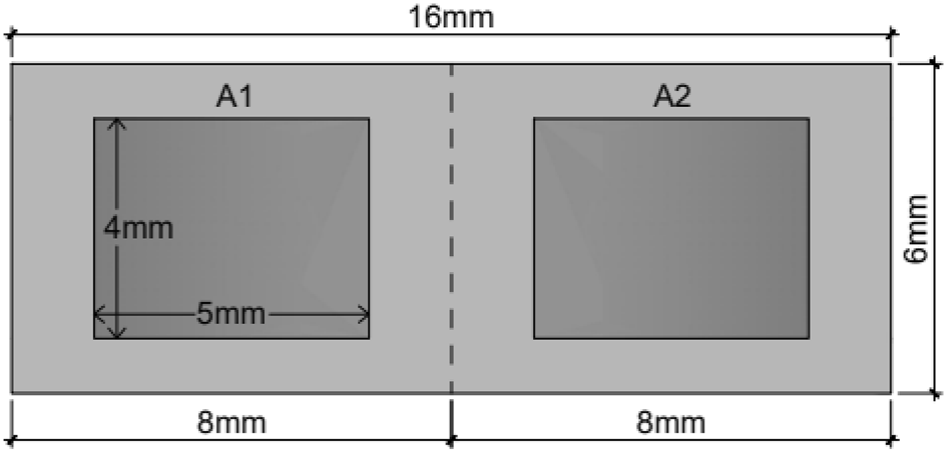
Dimensions of the specimens removed for grafting 16 mm (length) × 6 mm (width). Areas A1 and A2 were randomly selected for optical and SEM analysis, with dimensions of 4 mm (length) × 5 mm (width).

The obtained specimen was assessed for the presence of undesirable tissues such as adipose tissue. None of the three specimens exhibited macroscopic presence of excess tissues. The tissues were placed on cardboard paper for 1 min, and the mold was then used once again to divide them into two parts (anterior and posterior regions of the specimen), resulting in six sections measuring 8 mm (length), 6 mm (width) and 2 mm (thickness), as depicted in Fig. 6.

Three specimens from each mandible were fixed in a 10% buffered paraformaldehyde (formaldehyde) solution for 24 h to preserve the physical and chemical properties of the tissues and stored in 70% alcohol and stored in 70% alcohol in a refrigerator at a temperature of 4 °C according to the multi-user high-resolution microscopy laboratory (Labmic) protocol for tissue preparation for SEM. Subsequently, these tissues were processed and embedded in paraffin. Next, 3–5 mm sections were made, stretched onto histological slides, and stained with hematoxylin and eosin for histomorphological evaluation under optical microscopy.

The other three tissues were placed in a 2.5% glutaraldehyde solution and taken to the Labmic at UFG for critical point sample preparation, followed by analysis using SEM at the Regional Center for Technological Development and Innovation (CRTI) of UFG.

### Optical microscopy

The tissue specimens with dimensions of 8 mm (length), 6 mm (width), and 2 mm (thickness) were fixed in a 10% buffered formaldehyde solution for 2 h at pH 7.0 according to the Labmic protocol for tissue preparation for SEM. After fixation, the samples were processed using an automatic histotechnique device for dehydration, clearing, and paraffin embedding. Subsequently, the specimen was sectioned using a microtome (Leica RM2165), obtaining consecutive 5 μm thick sections from each block, which were placed on histological slides and stained using the Hematoxylin–Eosin (HE) method. These sections were used for the microscopic and histomorphometric characterization of the samples.

Image capture was performed using a digital camera attached to the optical microscope (Fluo Biológico Opticam O500R) on the 40× objective lens, calibrated adequately for histomorphometric analysis. Analysis of the slides was conducted using the OPTHD software (Opticam). Two photos of alternating fields were taken for each sample to provide the best image visualization for describing the tissue characteristics.

The optical microscopy analysis involved observing the types of cells present in each sample and the main tissue characteristics in the section under examination. This allowed for assessing the specimen’s surface, subepithelial region, and the base available on the slides.

### Scanning electron microscopy

#### Sample preparation for SEM analysis

Before performing SEM analyses, sample preparation is required to obtain good images. This preparation involves the processes of fixation, washing, dehydration, and drying of the samples, which are performed using a critical point apparatus utilizing carbon dioxide and metallization (this step was not performed, as it does not align with the research’s purpose and would compromise tissue integrity, affecting SEM analyses). Metallization makes the material conductive by depositing a metal, typically gold or platinum, onto the sample.

Sample preparation was performed following the tissue preparation protocol for SEM provided by Labmic. A 2.5% glutaraldehyde solution was used to store and transport the tissues to the laboratory, where the subsequent steps of sample preparation for SEM analysis were carried out. This solution was prepared using the following proportions: 30 ml of 25% glutaraldehyde, 150 ml of 0.1 M phosphate buffer, pH 7.2, 120 ml of distilled water, 0.1 M, pH 7.2, resulting in 300 ml of a 2.5% glutaraldehyde solution. This solution was stored in a refrigerator at a temperature of 4 °C for 24 h, and the incubation period was 2 h according to the laboratory protocol for tissue preparation for SEM. This was from each mandible from each group carried out in the School of Dentistry.

#### Sample preparation for critical point

**Figure 7:**
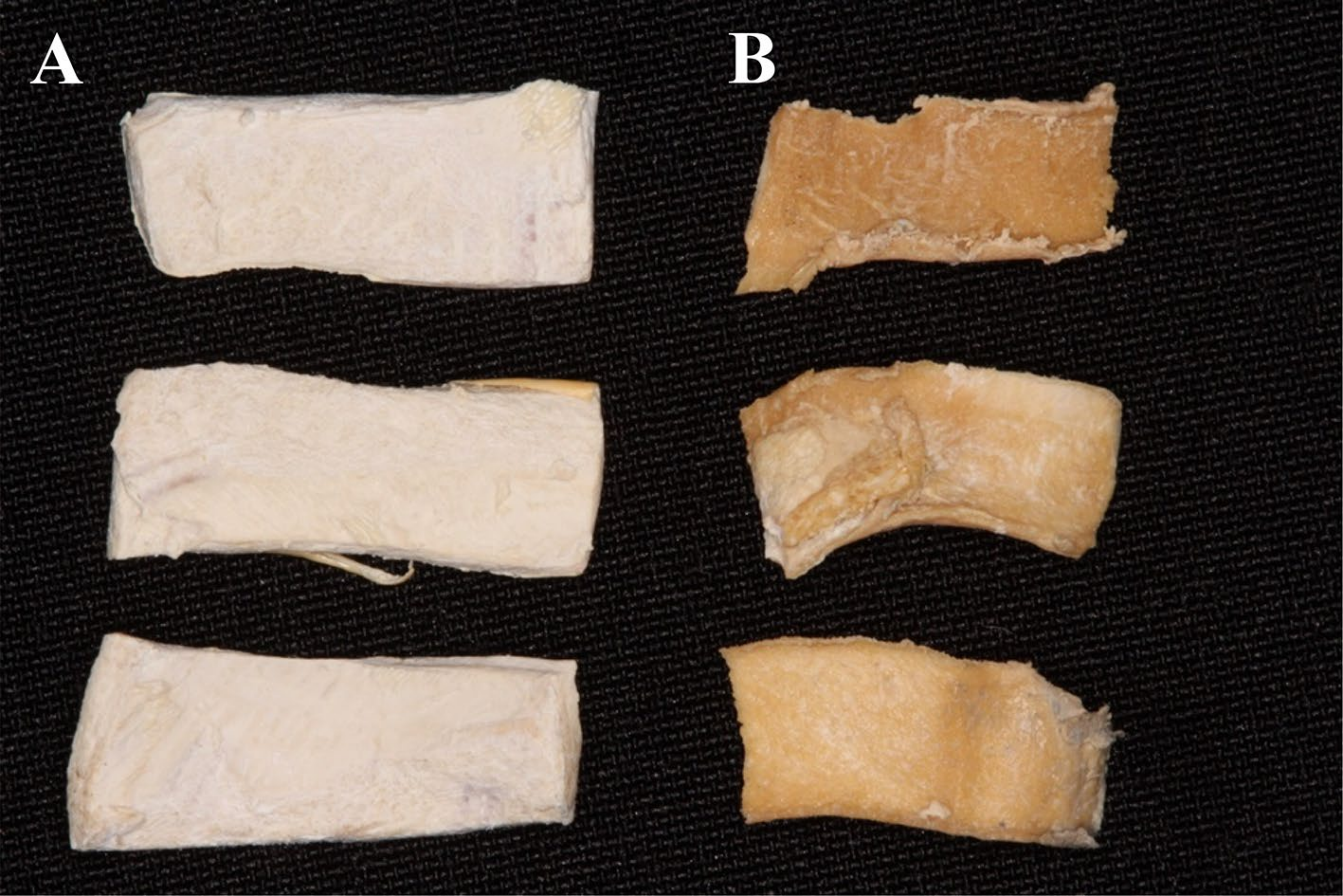
KMG samples after critical point preparation where the color of the specimens is different because of the thickness of each specimen removed with different techniques. (**A**) Samples removed with a scalpel; (**B**) samples removed with a mucotome.

After completing the prior steps of sample preparation, the final drying for SEM observation occurred under conditions with no transition meniscus of phases, thus avoiding the forces resulting from surface tension. Carbon dioxide was used as the exchange agent in the critical point drying (CPD) chamber. For each fluid, there is a characteristic temperature and pressure condition in which the liquid and gaseous phases of the fluid cannot coexist; this combination corresponds to the fluid's critical point. To reach the critical point, the samples, already fixed and fully dehydrated, were taken to the CPD chamber in a small volume of acetone. With the chamber isolated, liquid CO_2_ was injected. The chamber was at a temperature of 4–5 °C, and various substitutions were made until all acetone was removed. With the controlled chamber heating, CO_2_ became gaseous at a specific pressure without forming a transition meniscus and without causing a modification in the biological material’s structure. Despite the sample having undergone the same preparation process, the color of the specimens is different because of the thickness of each specimen removed with different techniques. (Fig. 7).

#### Scanning electron microscope (SEM) analysis

The specimens were analyzed using a Zeiss Axio Imager Z2 SEM (Zeiss, Jena, Germany) and an LSM700 laser scanning system equipped with a diode laser (405 nm). The scanning area size for each specimen was 400 × 500 µm, and the EC Plan-Neofluar 10×/0.3 HD M27 objective lens was used to obtain the best image quality for comparing the x (µm), y (µm), and z (µm) axes among the specimens. The acquired images were presented with topography assessment (Fig. 10A, B) and horizontal and diagonal 3D profilometry (Bruker, Germany).

For image capture and obtaining 3D profilometry values of the tissues removed by different techniques, the Zeiss Zen Black analysis and processing software (Zeiss, Jena, Germany) available at the Regional Center for Technological Development and Innovation (CRTI) of UFG was used.

SEM analysis involved assessing the peaks and valleys of the samples through horizontal and diagonal 3D profilometry. Two horizontal profilometries were conducted, dividing the sample into equal thirds, in addition to a diagonal profilometry. Surface topography data of the samples were obtained by calculating the percentages representing each topographical segment area relative to the total dimension of the evaluated specimen.

The digital profilometer, also known as a roughness tester, is a device that provides topographical characteristics of surfaces, with its three-dimensional (3D) image being more stable, reliable, and representative than two-dimensional (2D) images. It consists of many profiles in predefined areas, not just based on linear measurements, generating a 3D image from recorded data as a distribution of heights (peaks and valleys). This is done through specific software that provides mathematical parameters to identify the surface properties of the samples (rugosity)^33^. The surface roughness parameter (Sa) is based on the average standard deviation between peaks and valleys on a surface^35^.

#### Data collection and analysis

The data collected after analyzing the samples using scanning electron microscopy (SEM) and optical microscopy were assessed, related, and described.

#### Waste disposal

Following the KMG removal procedures, the porcine hemi-mandibles were disposed of in accordance with Resolution No. 306/2004 of the Ministry of Health, National Health Surveillance Agency (ANVISA). The disposal of hemi-mandibles and other waste was carried out based on the type of waste and its classification group: the mandibles were discarded as Group A waste (potentially infectious waste); the 2.5% glutaraldehyde and 10% paraformaldehyde solutions were discarded as Group B waste (chemical waste); papers, plastics, packaging were discarded as Group D waste (common waste); and the used scalpel blades were discarded as Group E waste (sharps waste).

#### Risk and benefit analysis

Some of the potential risks associated with the study included accidents with sharp instruments, chemical compound accidents, and a lack of adherence to animal research guidelines. These risks were minimized through using full personal protective equipment (PPE) by professionals, training, and adherence to all guidelines and regulations prescribed by the Ethics Committee on Animal Use (CEUA) of UFG.

### Ethical considerations

An experienced and trained dentist carried out the procedures. The specimen removal procedures with the scalpel and mucotome were conducted at the Research Center for Prosthesis and Implants (NPPI) of the School of Dentistry at the Federal University of Goiás (UFG). This work began only after approval and issuance of the substantiated opinion by the CEUA of UFG.

## Results

### Optical microscopy

**Figure 8:**
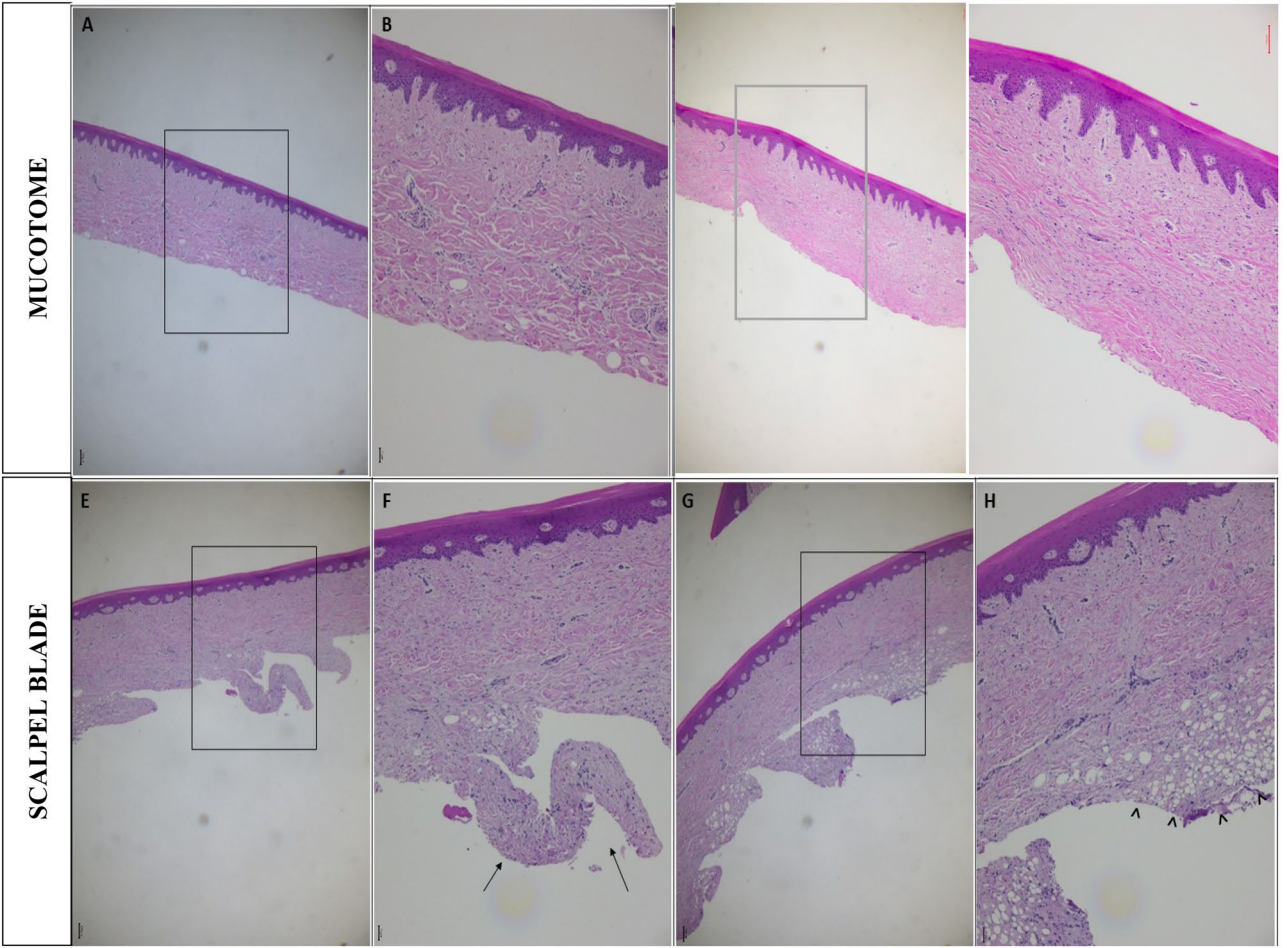
Images obtained through optical microscopy, objective lens, magnification ×5 and ×40. Grafts removed from porcine mandibles with a mucotome (upper) and scalpel (lower) stained with hematoxylin and eosin. (**A**) Mucotome, objective lens, magnification ×5; (**B**) mucotome objective lens, magnification ×40; (**C**) mucotome, objective lens, magnification ×5; (**D**) mucotome, objective lens, magnification ×40; (**E**) scalpel, objective lens, magnification ×5; (**F**) scalpel, objective lens, magnification ×40, arrow symbol: irregularities at the base of the tissue; (**G**) scalpel, objective lens, magnification ×5; (**H**) scalpel, objective lens, magnification ×40 hat symbol: adipose tissue cells.

The images of the slides below were obtained through optical microscopy. Three slides of tissue removed with a scalpel and three slides of tissue removed with a mucotome were made. In Fig. 8, two slides are shown from tissue removed by scalpel (A–B and C–D) and two from tissue removed by mucotome (E–F and G–H). The most representative slides were chosen and shown in Fig. 8. The presence of stratified squamous keratinized epithelial tissue and dense modeled connective tissue can be observed in the tissue slides removed with the mucotome and scalpel (Fig. 8). In the tissue removed with the mucotome, a linearity can be seen at the base of the tissue and in the overall tissue thickness, submucosa presence, fibrous connective tissue ideal for graft success (Fig. 8A, B). In contrast, tissue removed with the scalpel shows more irregularities, deepening during tissue removal and significant irregularity at the graft base (Fig. 8E, F). Moreover, undesirable tissues, such as adipose tissue, can be noted (Fig. 8G, H).

Microscopically, tissues removed with the mucotome exhibit more favorable characteristics, expected to ensure a higher chance of graft success. However, for fragile regions, the mucotome is limited in removing undesirable tissues due to its high cutting power. Therefore, the operator must control the force applied when handling the mucotome. Conversely, tissues removed with a scalpel exhibit limitations regarding greater irregularity at the tissue base, depth of removed graft, and the presence of undesirable tissues.

### Scanning electron microscopy

**Figure 9:**
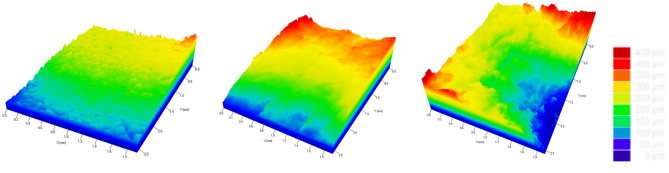
Surface profile composed of shape and roughness for mucotome samples 1, 2 and 3.

Two areas per sample (a and b) with dimensions of 1920 × 2560 μm (x and y axes) were analyzed. The obtained images and the profiles were collected at two points on each axis (x and y). The following figure are of the samples removed with a mucotome and demonstrate the topography of the analyzed KMG samples by SEM and 3D profilometry in both diagonal and horizontal directions in various areas. As observed in the figures the “ramp” effect occurs due to the angle and pressure applied by the mucotome on the mandible (Fig. 9).

**Figure 10:**
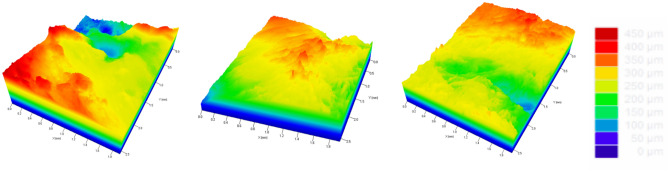
Surface profile composed of shape and roughness for scalpel samples 1, 2, and 3.

The Fig. 10 are of the specimens removed with a scalpel and demonstrate the topography of the KMG samples analyzed by SEM and 3D profilometry in diagonal and horizontal areas. The “ramp effect” was not observed in this figure, resulting in larger grafts with a greater presence of connective tissue (Fig. 10).

**Figure 11:**
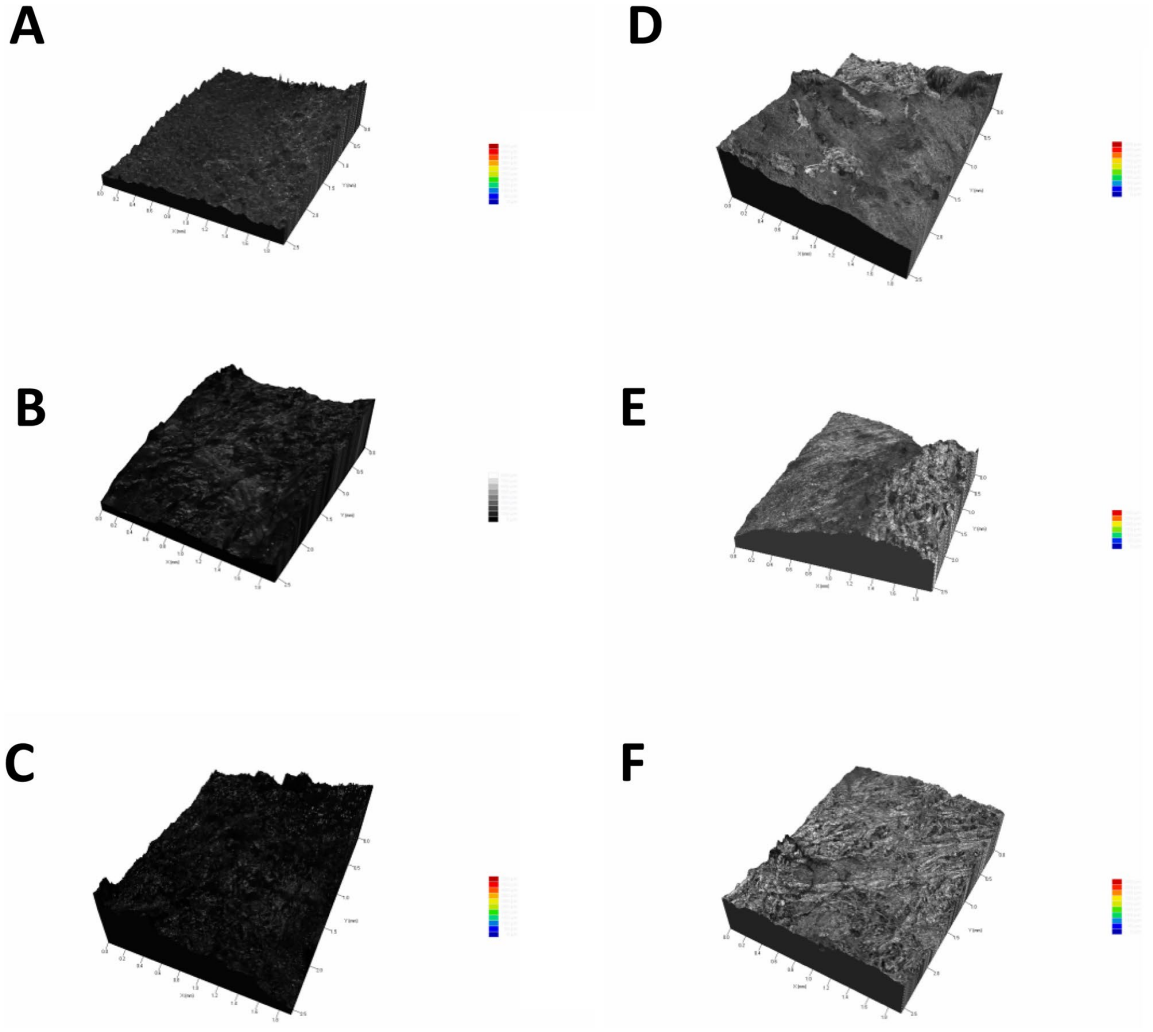
Surface profile composed of shape and roughness (monochromatic). SEM comparison. (**A**–**C**) samples removed with a mucotome, and (**D**–**F**) are from samples removed with a scalpel.

The Fig. 11 depict the topography of the KMG samples analyzed by SEM and 3D profilometry in diagonal and horizontal areas.

Figure 11A–F revealed differences in the surfaces of the samples measured by SEM. These differences occurred due to the chosen method for tissue removal. The surfaces of samples removed with a scalpel exhibit more irregularities compared to the surfaces of samples removed with a mucotome. A common surface pattern can be observed among the samples removed with a scalpel, with a significant presence of crests and valleys throughout the sample’s dimensions. In contrast, the samples removed with a mucotome exhibit greater uniformity across all analyzed samples, with the almost absence of significant crests and valleys.

**Figure 12:**
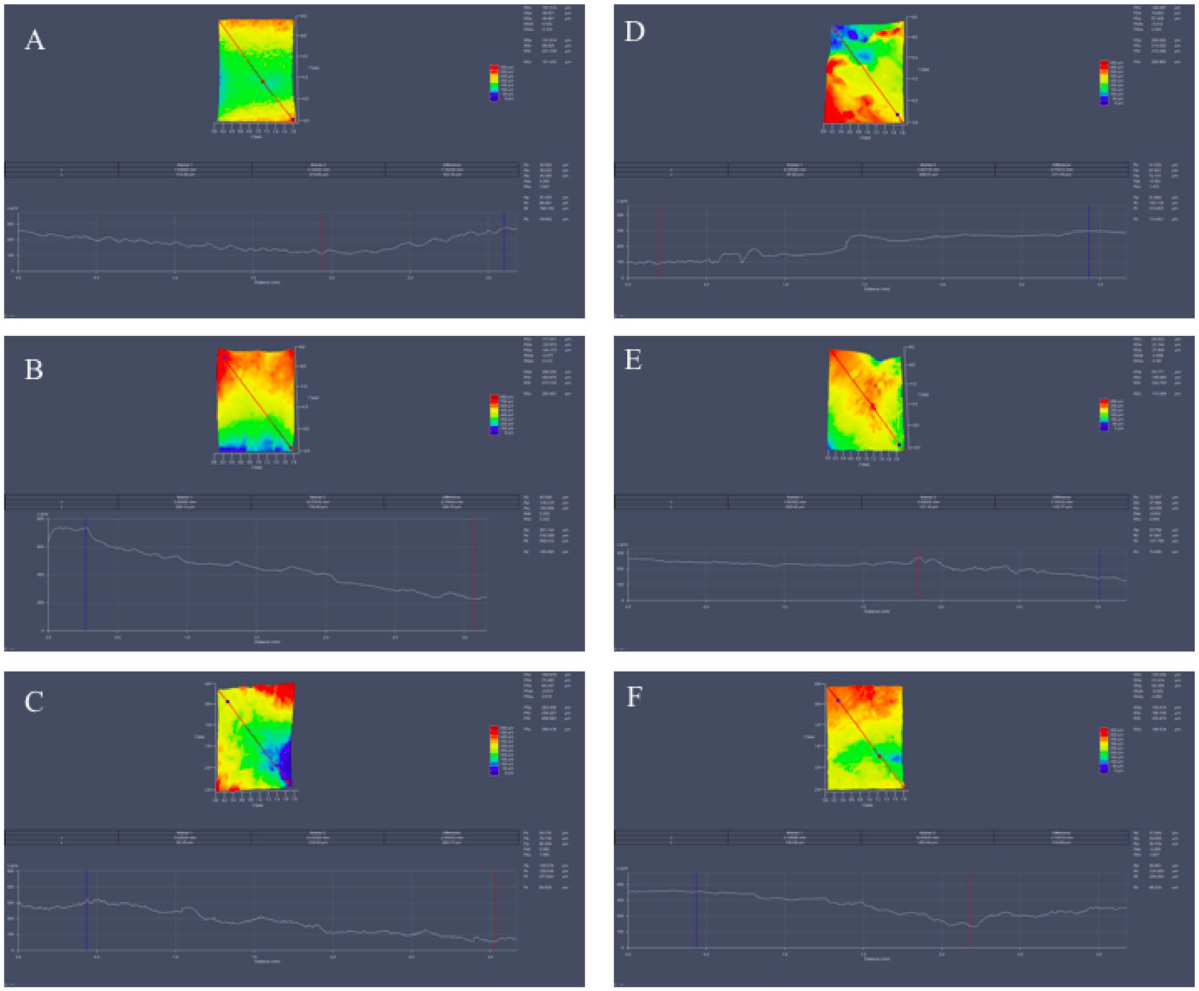
3D diagonal profilometric analysis of samples 1, 2, and 3 with a mucotome (**A**–**C**) and 1, 2, and 3 with a scalpel (**D**–**F**).

The Fig. 12 allow for the observation of the diagonal topographical variation of the analyzed samples. When examining the graphical representation of the topography on the diagonal plane of tissues removed with a scalpel, a greater variation is noticeable compared to the graphical representations of the topography of tissues removed with a mucotome.

**Figure 13:**
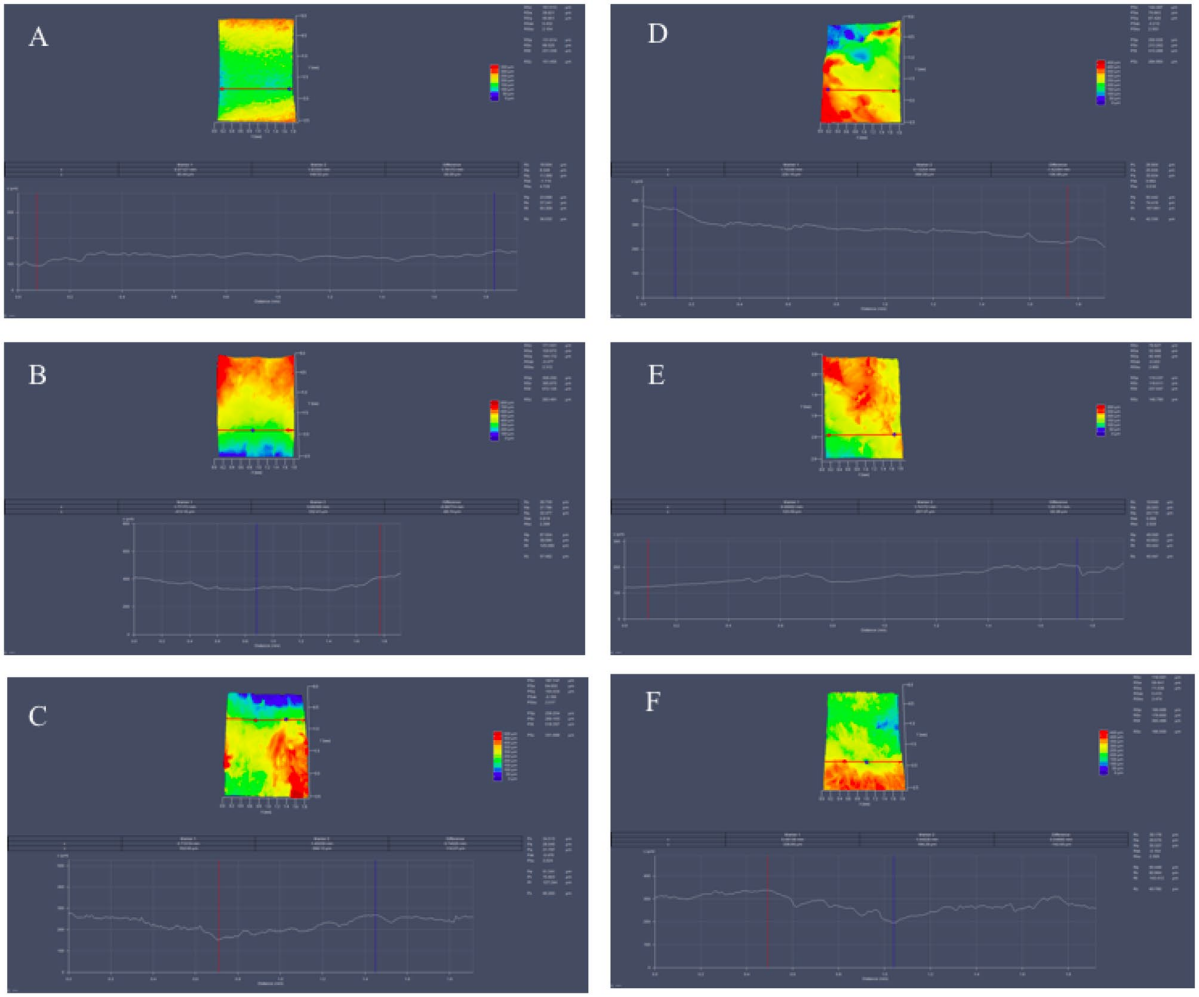
3D horizontal profilometric analysis of samples 1, 2, and 3 with a mucotome (**A–C**) and 1, 2, and 3 with a scalpel (**D–F**).

Figure 13 allows for the observation of horizontal topographic variation in the analyzed samples. When examining the graphical representation of the surface in the horizontal plane for tissues removed with a scalpel, greater variation is noticeable when compared to the graphical representations of the topography of tissues removed with a mucotome.

The surface roughness values of each sample obtained through 3D profilometry are described in Figs. 12 and 13. The average of the values obtained was performed by the 3D digital profilometer software in the vertical and horizontal direction of the samples, these being: mucotome vertical surface roughness = 19,133 μm; mucotome horizontal surface roughness = 21,948 μm; scalpel vertical surface roughness = 193.269 μm; scalpel horizontal surface roughness = 202,489 μm.

## Discussion

The results of the analyses in optical microscopy and SEM allow us to infer that the graft removal technique using the mucotome enables the removal of significantly more regular grafts compared to the conventional scalpel technique. Furthermore, tissues removed with the mucotome exhibited a smoother, more uniform surface without undesirable cells, such as adipose tissue or other cells.

According to Maurer et al.^36^, the submucosa of adipose tissue and epithelial tissues should be removed entirely, especially in cases of subepithelial connective tissue grafts, to prevent complications and to allow the organism to induce the formation of new keratinized tissue. In this study, KMG was used, and samples removed with a scalpel exhibited the presence of adipose tissue in large areas. In contrast, the samples removed with the mucotome did not show any presence of adipose tissue. This distinction is due to the thin (0.5 mm) and uniform cuts the mucotome allows during graft removal.

Thoma et al.^37^ conducted a systematic review and concluded that autogenous grafts statistically increase the width of the attached gingiva. Using the mucotome in future clinical research will enable the evaluation of whether there is a clinically significant increase in gingival width using this technique compared to the scalpel technique.

Although it has been used for many years, the method of removing gingival grafts using a scalpel blade has limitations regarding surface irregularity and the presence of undesirable tissues that may contribute to graft failure in the recipient area ^4,30,32^. To address this, we sought to control variables related to graft removal and specimen preparation during the development of this study. Porcine mandibles were chosen due to tissue availability and ease of working with these specimens.

Tarasenko et al.^38^ conducted a controlled and randomized study and comparatively analyzed methods for increasing keratinized tissue, concluding that EGL was the most effective technique for increasing peri-implant keratinized tissue. In this study, we chose to maintain KMG samples intact as they were removed from the porcine mandibles to avoid compromising the quality and enable the most faithful analyses possible. This allowed us to observe the presence of epithelial tissue in all samples.

Dong, Sullivan, and Stout^39^ describe that 3D surface analysis has been prominently emphasized by the industry and academic institutions because it provides a better representation of the surface. The surface topography of samples is often described through two-dimensional profiles (2D), where roughness (Ra) typically refers to a profile or the average of two to three profiles^40–42^. However, according to Hutchings^43^ and Whitehead et al.^44^, 3D profilometry allows area studies. It is capable of reading various profiles on the same surface, allowing the collection of data with minimal variations at the nanometer scale. Based on these studies, the Digital 3D Profilometer was used, and six profiles were performed for each specimen.

Different methods are described in the scientific literature for characterizing surface roughness. Still, the most accepted method determines the profile along a line on the sample’s surface using a mechanical trace and expresses roughness through the undulations of this profile^45^. In this study, profiles were traced in three different areas for diagonal and in three different areas for horizontal of the samples. They showed less variation in samples removed with the mucotome. The most representative images were chosen and shown in Figs. 12 and 13.

Among the samples analyzed using 3D profilometry, those removed by the mucotome technique exhibited lower roughness than those removed with a scalpel and less variation has been observed in the graphs of samples removed by the mucotome technique compared to the other technique, contributing to the validation of the study’s initial hypothesis.

Through the evaluation of tissues using optical microscopy, we were able to reinforce the hypothesis that tissues removed by the mucotome technique have a more uniform surface and better tissue quality than tissues removed by the conventional technique, which exhibited undesirable cells and greater irregularities. However, bone cells associated with tissues removed with the mucotome can be observed. This may have occurred due to the pressure applied with the mucotome on the mandible and the thin phenotype presented by porcine mandibles.

It is suggested that the removal of these grafts can be carried out using the mucotome to obtain thinner grafts with a regular surface and a lower chance of undesirable cells, ensuring a more favorable healing process and, consequently, a higher success rate in surgical graft procedures.

Individuals with a thin gingival phenotype and a narrow band of keratinized tissue are prone to developing gingival recession^46^. To correct these defects^47^, removed subepithelial connective tissue grafts using a mucotome and obtained uniform grafts with thicknesses between 1 and 1.5 mm due to the removal technique from the donor site, demonstrating success in healing and coverage of the recipient site. This corroborates with the results of the present study, demonstrating that graft removal using the mucotome may be advantageous compared to the conventional technique.

Upon analyzing the images from optical microscopy and SEM, it is evident that the results consistently favor tissues removed with the mucotome. This technique has proven advantageous in minimizing shortcomings, exhibiting reduced dependence on the operator’s experience and skills, and offering greater precision, standardization, predictability, and practicality during graft removal. As a result, patients can feel more secure when opting for this technique and are likely to experience a more favorable post-operative outcome. In addition to enabling more precise incisions compared to the conventional scalpel technique, another advantage of refining the method using the mucotome is its capability to remove tissues that are 1–2 mm thinner than grafts removed by the conventional manual scalpel technique^48^. Thinner grafts can survive longer without a blood supply until neovascularization occurs, leading to integration and subsequent healing of the recipient area, thus ensuring a higher likelihood of surgical procedure success^49^.

Moreover, the mucotome presents itself as equipment that provides a novel approach to graft removal, offering greater ease during execution and increased predictability during the pre- and post-surgical phases ^47–49^. Prestin et al.^50^ conducted a study to measure oral cavity tissues and found that the epithelium of the hard palate has an average thickness of 239 µm. This area is typically the preferred region for removing soft tissue grafts in humans. In the present study, it can be observed that porcine mandibles have thin tissues that may complicate their removal, representing a potential limitation of this study. However, favorable results were obtained when using the mucotome on these tissues, which proved superior to the conventional technique. Therefore, it can be inferred that when performing the removal of KMG in human tissues, which are generally thicker, the chances of success are even higher and more advantageous, in addition to being easier to remove compared to the scalpel technique. Other potential limitations pertain to the cost of the mucotome, the vibration, the noise generated by this device, and the inability to adjust the graft removal thickness for customization in each case.

The primary purpose of this study was to serve as preliminary research for future clinical investigations that can compare graft removal techniques and assess their advantages and limitations such as despite the lack of a statistical analysis, we would like to highlight that the comprehension of the article and the richness of the presented results were not impaired. This is attributed to the exceptional quality of the collected data and the descriptive approach, which included the use of diagonal and horizontal 3D profilometric images, expressed clearly through line graphs, highlighting the surface roughness of each sample. We believe these elements strengthen the robustness and relevance of our work to the scientific community. Furthermore, future research may evaluate the healing of both donor and recipient regions of the graft and post-operative pain sensitivity. The results are promising and align with scientific knowledge and clinical practice.

## Conclusion

When KMG grafts are removed using the mucotome technique, they exhibit greater uniformity and a reduced presence of undesirable cells compared to the scalpel technique. The removal of KMG grafts can be performed using the mucotome to obtain thinner and more uniform grafts, increasing the chances of success in soft tissue graft surgical procedures. Well-designed clinical studies are needed to compare the two techniques for KMG graft removal in human tissues, assess the clinical relevance of mucotome use, and evaluate variations in success rates between the two techniques.

## Data Availability

All data generated or analyzed during this study are included in the article and its supplementary information files. Source data are provided with this paper.
